# Comparing the Efficacy of Static and Dynamic Graph Types in Communicating Complex Statistical Relationships

**DOI:** 10.3389/fpsyg.2019.02986

**Published:** 2020-01-17

**Authors:** Jeffrey Chase Hood, Cade Graber, Gary L. Brase

**Affiliations:** Department of Psychological Sciences, Kansas State University, Manhattan, KS, United States

**Keywords:** graphs, data interpretation, main effects, interaction effects, graph design

## Abstract

Graphs are useful tools to communicate meaningful patterns in data, but their efficacy varies considerably based on the figure’s construction and presentation medium. Specifically, a digital format figure can be dynamic, allowing the reader to manipulate it and little is known about the efficacy of dynamic figures. This present study compared how effectively static and dynamic graphical formats convey relationship information, and in particular variable interactions. Undergraduates (*N* = 128, 56% female, *M*_age_ = 18.9) were given a brief tutorial on main effects and interactions in data and then answered 48 multiple-choice questions about specific graphs. Each question involved one of four figure types and one of four relationship types (main effect only, interaction only, main effect and interaction, or no relationship), with relationship types and graphical formats fully crossed. Multilevel logistic regression analysis revealed that participants were fairly accurate at detecting main effects and null relationships but struggled with interaction effects. Additionally, the static 3D graph lowered performance for detecting main effects, although this negative effect disappeared when participants were allowed to rotate the 3D graph. These results suggest that dynamic figures in digital publications are a potential tool to effectively communicate data, but they are not a panacea. Undergraduates continued to struggle with more complicated relationships (e.g., interactions) regardless of graph type. Future studies will need to examine more experienced populations and additional dynamic graph formats, especially ones tailored for demonstrating interactions (e.g., profiler plots).

## Introduction

The information processing limitations of the human brain make unaided interpretations of large datasets impractical. This is particularly problematic in science where researchers attempt to identify trends, covariances, and interdependences within large sets of data in order to gain insights about variables of interest. Quantitative, theoretically driven research requires effective ways to meaningfully consolidate and interpret data. One common way to simplify the complexity of data is through graphical representation (graphs). However, there is no consensus on a “best” way to graph data and plenty of evidence of frequent misinterpretations of graphs and figures. This research investigates the efficacy of various graph formats, specifically including both simple and more complex relationships between variables and including graph formats beyond traditional print representation (e.g., interactive figures).

The purpose of graphical data representations is to condense the information in the data set, and in particular the relevant properties (e.g., trends or covariations), while faithfully maintaining the integrity of the overall dataset representation. This can aid the researcher both in data analysis and in the communication of results. However, not all graphs achieve these goals. Mistakes in the transmission of information via graphs can either be a consequence of presentation error, in which false or misleading information is depicted (i.e., lying with statistics) or they can be the result of misinterpretation of a “correct” graph on the part of the reader. The willful production of false or misleading graphs is a matter for ethical discussions. The present research is concerned with the ability to interpret faithfully presented graphs with different constructions.

The following subsections briefly describe some known influential considerations in graph construction and the necessity of certain graph elements. This leads to the issues associated with multidimensionality; how one should graph data that co-vary in more than two dimensions (i.e., two-way and higher interactions). Higher-order relationships are, by their nature, more complex and difficult to comprehend. As such, difficulty of building effective graphs increases, but so does the utility of graphs to facilitate the interpretation of these relationships.

### Principles of Effective Graphs

A seminal review of the essentials of effective graph design comes from Kosslyn (2006), which includes eight fundamental principles that will be used in this paper. The first two of these principles are the principle of **Relevance** (graphs should have no more and no less information than necessary to convey the intended message) and the principle of **Appropriate Knowledge** (graph efficacy is contingent on the appropriate prior knowledge of the reader). The next two principles are those of **Salience** (the greatest perceptible differences in a graph should direct the reader to the most relevant components) and **Discriminability** (meaningful differences should differ by large enough margins to be visually distinguished; e.g., see supporting research by Hollands and Spence, 2001). The next two principles are the principle of **Compatibility** (the information format should map intuitively onto the intended message; e.g., see supporting work by Gattis and Holyoak, 1996) and the principle of **Information Changes** (the graph display should remain constant to intuitively signify unchanging information, and should change to signifying that the information is changing). The final two principles rest a bit more on the inherent mental characteristics of people’s memory and visual cognition. The principle of **Capacity Limitations** says that graphs should not ask people to balance more than about four pieces of information simultaneously, due to the limitations of human working memory capacity (Cowan, 2010; see also work by Meyer et al., 1997). Lastly, the principle of **Perceptual Organization** says that graphs should utilize the tendencies of the visual system to group objects by their proximity, orientation, and visual similarity to each other.

Early research, naturally, focused on more straightforward and clear possible implications of these principles (e.g., graphs showing a simple difference between two means or a single correlation). Traditional graphing methods, if they follow the recommendations detailed above, are well-suited to portray single effects. A difference between two groups could, for example, easily be illustrated in a graph of two columns (Principle of Relevance), and a large effect should be reflected in the difference between the two columns being easily discriminable (Principle of Discriminability). Furthermore, it is relatively easy to make the bars prominent against a plain background (Principle of Salience), and visually similar in shape and color (Principle of Compatibility). Such a graph also proffers only a few pieces of information (per the Principle of Capacity Limitations).

Many research findings, however, are more complex and nuanced than the basic example described above. A simple finding often leads to further research that branches and narrows, with potential moderating and mediating factors or other complications. As the research shifts to these more contingent relationships there is a need for graphical representations that can clearly and effectively portray those complex situations.

### Multidimensionality in Graphs

How data are portrayed graphically should reflect the information those data represent. One would not reasonably use a line graph to depict the proportion of a population that likes lemon meringue (a pie chart would surely be better for this). The challenge that arises when the information is complex, multivariate, and involves interactions is to have a graph portrayal that faithfully and effectively conveys those relationships.

As a starting point, suppose we have data that includes a simple categorization of people by sex (i.e., male or female) that can be denoted by two numbers, and also people’s height that can be defined on a numerical scale. All of the information about each person is contained in a simple pair of numbers so far: *x* (sex) and *y* (height). Every unique pair of *x* and *y* values in this sample could then be plotted on a Cartesian plane and the result would be a scatterplot. If one plots the averages of the *y* values (height) for both categories of *x* (sex) and draw bars from the *x*-axis up to those points, then the result is the traditional bar graph described earlier. If one replaces the bars with a line connecting the two average values, then the result is an illustration of the bivariate correlation between the two variables. Regardless of the graphical format, one can see that at the gross level of examination the number of dimensions used to display information is equal to the number of dimensions used to define each participant. The inclusion of additional variables to the description of the individual therefore complicates the graph construction.

What happens when we extend this example by incorporating a third variable? Whereas before each participant was defined only by a score on *x* (sex) and *y* (height), now each can additionally be described on a third dimension, *z*, that could signify the participant’s age. The analysis must consequently be expanded, as the inclusion of this second predictor variable increases the number of potential effects from one to three: the main effect of sex on height, the main effect of age on height, and the interaction effect of sex and age on height. After examination of the two main effects, one sees that age is positively correlated with height and that males are taller on average than females. However, both of these effects are qualified by the contingency between them (i.e., their interaction). While males are on average taller than females at maturity, females tend to reach their full height at an earlier age than males. In this example, age acts as a moderating variable and describes why, during adolescence, it not uncommon that males tend to be shorter than females of the same age, a detail lost in the earlier, simpler example.

**FIGURE 1:**
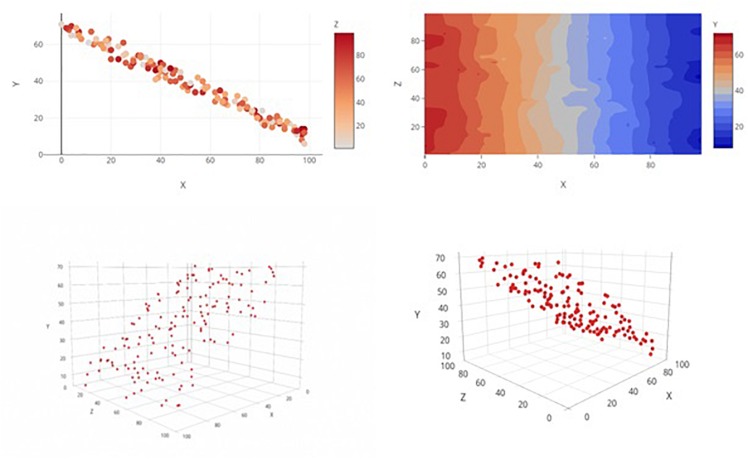
Examples of the four graph types, each displaying the same main effects(s) only relationship. Clockwise from top left: 2D Color Plot, Contour Plot, 3D Rotatable Plot, and 3D Static Plot.

For the graphs described thus far, the information can be displayed in a two-dimensional (2D) format without violation of Kosslyn’s principles. However, this is only because one of the predictors in this scenario, sex, is categorical. Commonly, three or more of the variables of interest are continuous. In such cases a researcher faces the choice of either subdividing the continuum into sections (categorize by section in order to simplify analyses) or retaining the full continuum. The subdividing tactic is very common in psychological research (Young, 2016), but research suggests that maintaining the continuous integrity of one’s data is almost always the preferable choice. This is because the categorization of continuous data unnecessarily reduces the power of the study and masks underlying contingencies/non-linearities, the discoveries of which may require an intact continuum (Young, 2016). One way to resolve the conflicting objectives of clear graph communication is to explore some more advanced graph options. Three or more continuous variables are difficult to present and interpret graphically in traditional 2D formats that impose visuo-spatial constraints. The current research examined the efficacy of four different graph formats in displaying different types of relationships between three continuous variables (see Figure 1).

#### Two-Dimensional Scatterplot With Delineating Color Bar

The top-left graph in Figure 1 shows a 2D *x*-*y* scatterplot and allows the third variable to vary as a function of color. Color is easily displayed along a continuum for color (a color scale) that can be consulted alongside the scatterplot of data points, allowing for more immediately accurate observations of each data point’s level on the 3rd dimension. This method is compatible with all color-enabled print and digital media, but it does not involve any interactive elements for user manipulation of graphs.

#### Color Contour Plot

Though strictly for displaying general trends/relationships among variables rather than the entire set of individual data points, a color contour plot (top-right in Figure 1) is a natural extrusion of the 2D scatterplot of predictor variables into a third dimension (color). The result is a topographical surface that denotes how a region’s “height” (the level of the dependent variable, coded by a color continuum) is differentially affected by various combinations of the values of the two predictor variables (i.e., “height” fluctuates across graphical regions). This method may permit a more intuitive interpretation of trend than that of the above method, given that *both* predictor variables are assigned to the 2D space while the dependent variable is afforded its own, unique dimension. Indeed, there is evidence that interpretation accuracy depends upon the assignment of the variables to either the *x*-axis or legend space (Ali and Peebles, 2013). This method also requires color-enabled media but does not make use of interactive elements.

#### Three-Dimensional Static Graph in Perspective

The third graph type (Figure 1, bottom-left) is a 3D scatterplot on which the three continuous variables are plotted along the *x*-, *y*-, and *z-*axes. This image is static and is “tilted” at an angle to give the illusion of depth perception. This perspective mimics the visual sense of depth and should facilitate discrimination of distances and relationships among data points in a graph. By nature, a 3D graph requires observers to make use of such depth cues as occlusion, proximity, and gridline reference (Dosher et al., 1986). However, consigned to a single static viewpoint, the observer may have trouble resolving the overall trend depending on how much variability is present and how well any one particular viewpoint can capture it. Even though this method may pose a more viable option to traditional outlets that lack color-enabled media, the loss of informative function is costly.

#### Three-Dimensional Rotatable Graph in Perspective

The last graph type (Figure 1, bottom-right) is a rotatable variant of the 3D perspective graph and takes advantage of digital publishing’s capability to mitigate some of the weaknesses of the static variant. With this version readers can utilize cues such as motion parallax to better judge the distances and relative positions of data points as they rotate the graph. However, this method’s ostensible functional advantages over static graphs are hampered by its compatibility shortcomings; only certain types of media (e.g., interactive digital outlets) will be able to employ it.

The 3D rotatable graphs are by far the most versatile of these four options in terms of functionality; they display all variables along three principle axes and so avoid the relegation of any variable to a symbolic space like a legend (Principle of Capacity Limitations). The intuitive correspondence between a number line and a continuum of numerical data also, in principle, makes the changes in values of each variable easier to grasp (Principle of Compatibility). Inasmuch as humans are biologically accustomed to localizing and tracking objects through 3D space, people should be very capable of discerning relationships among those data points (Principle of Perceptual Organization). The complication for the 3D rotatable graph is that it runs up against the limitations of traditional (2D paper) media.

Two-dimensional color graphs are currently common for several reasons: they are easily constructed, similar to bar and line graph formats (Principle of Appropriate Knowledge) and the overall relationship between the *x*- and *y-*axis variables can be quickly gauged. Ali and Peebles (2013) found that people perform well in discerning the relationship between the dependent *y* variable and a legend-bound *z* variable delineated by color. Interactions between the two independent variables, however, may present a greater difficulty for 2D color graphs than for the 3D rotatable format. It is not immediately clear, for instance, how the effect on *y* of any specific *x* value would be modulated by color shifts (the representative *z* value), or vice versa. Indeed, unless the observer knows to scrutinize specific patterns (“twisting” or “spreading” in the data points), she may be at a loss. It should also be noted that inspection of spatial patterns alone does not give one much insight into the specifics of an interaction. One must attend to color changes *while* also attending to spatial patterns if one is to discover anything of import about the interaction, and this can overtax one’s working memory (Principle of Capacity Limitations).

Although lacking actual rotatable functionality, the 3D static graph has been utilized in research media (e.g., Khemlani et al., 2012). The features of a 3D rotatable graph (described above) similarly apply to the static version, except the critical element of rotation functionality. This one missing element, though, can critically hamper an observer’s understanding of data sets containing important interactions that cannot be orientated in such a way that guarantees the visibility of all relationships and interactions (Principle of Salience).

There is relatively little precedence for 2D contour plots in the psychological literature. There is, however, reason to believe that the discernibility of main effect relationships is differentially affected by the assignment of the independent variables to either the axis or the legend (Ali and Peebles, 2013). From this, it is not unreasonable to assume that a similar phenomenon may exist for the assignment of the dependent variable. The unconventionality of this graph type, however, may impair its being accurately interpreted.

### Hypotheses

Two hypotheses guided the following research. The **first hypothesis** is that there will be an effect of graph type on accurate interpretation, with average accuracy being, from best-to-worst: 3D rotatable, 2D color, 3D static, and 2D contour. This hypothesis is consistent with Kosslyn’s principles, Ali and Peebles (2013), and most people’s intuitions. The **second hypothesis** is that there will be an effect of relationship type on accurate interpretation, with accuracy being, from best-to-worst: no relationship, main effect(s) only, interaction only, and main effect(s) with an interaction. This order follows directly from the increasing level of complexity across these four types.

## Method

### Participants

A total of 179 undergraduate students enrolled at a state university in the Midwest participated in the study. Of these, responses from 51 participants were dropped due to failing *a priori* exclusion criteria (38 were removed for completing the study in under 5 min, two for completing less than 75% of the study, and 11 for answering correctly at or below chance levels, all indicators of low-quality responding). Therefore, data from 128 participants (56% female, *M*_age_ = 18.9) were analyzed. All participants were recruited from a general psychology course that required participation in their choice of research studies at the university. This study was carried out in accordance with the recommendations and with the approval of the researchers’ Institutional Review Board (IRB).

### Design and Materials

**FIGURE 2:**
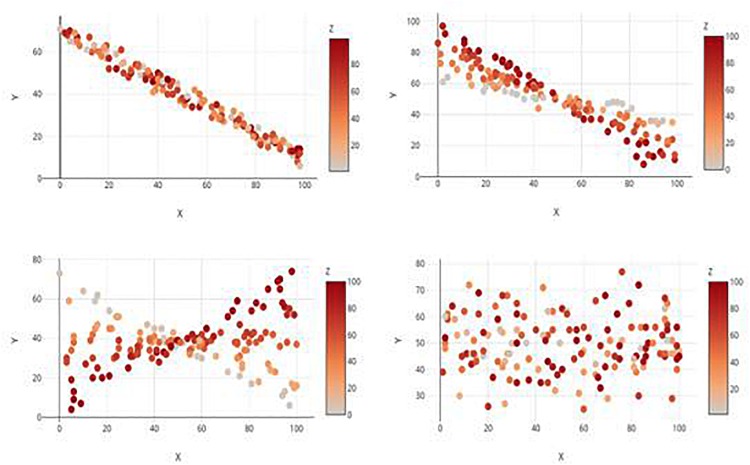
Examples of the four relationship types, each displayed by a 2D Color Plot. Clockwise from top left: Main Effect Only, Main Effect with Interaction, no Effects, and Interaction only.

Participants all completed a randomized series of graph interpretation tasks, followed by basic demographic questions (age, sex, education, major, and standardized test scores). The graph interpretation task consisted of 48 different graphs that varied by type and depicted relationship, but that all exhibited three continuous variables labeled *x*, *y*, and *z*. For the sake of simplicity, participants were instructed to treat *y* as the dependent variable and *x* and *z* as the two independent variables. Each graph was one of four possible types (2D Color Plot, 2D Contour Plot, 3D Static, or 3D Rotatable; see Figure 1) and depicted one of four possible relationships:(1) only main effect(s); (2) only an interaction; (3) main effect(s) and an interaction; and (4) no main effects and no interaction (see Figure 2 for examples). In the 2D contour plot, the *x*-axis was the horizontal axis, the *z-*axis was the vertical axis, and the *y-*axis was a vertically presented color gradient located to the right of the graph. In the other three graphs, the *x*- and *y-*axes denoted the horizontal and vertical axes, respectively, while the *z-*axis denoted an axis orthogonal to the first two in the 3D graphs and a color gradient in the 2D color plot graph similar to that of the contour plot. This effectively split the stimuli into 16 type/relationship pairings, and three distinct sets of randomly generated data were utilized across the graph types for each of the four relationships, resulting in a total of 48 tasks. All stimuli were created using the Plotly graphing website^1^. The study design thus was a repeated-measures experiment (per the criteria in Keppel, 1991; Shadish et al., 2002; Rosenthal and Rosnow, 2008).

### Procedure

The survey was presented through an online survey software (Qualtrics) and included an informed consent process and instructions about the nature of the task. Prior to the actual tasks, participants were shown a tutorial page with definitions for “main effect” and “interaction”, followed by two examples (one containing only a main effect and one containing a main effect and an interaction). The examples displayed the data sets in each of the four representative graph types and were accompanied by a brief explanation of how one could go about interpreting the correct relationship.

During the test itself a small key containing shorter definitions of the terms “main effect” and “interaction” was located above each graph so that participants could reference these during the test. Graphs were presented one at a time in conjunction with four response options (the four possible relationships), from which participants were told to choose the option that best described what they saw in the data. Afterward, participants supplied demographics information and were debriefed.

## Results

The data were analyzed using multilevel logistic regression in order to model the within-subject dependencies inherent in repeated-measure data and to appropriately account for the dichotomous nature of the outcome variable (i.e., correct or incorrect). When using multilevel modeling, it is important to determine the appropriate random effects structure for the model before analyzing any fixed effects. To do this, the Akaike information criteria (AIC) of three models were compared to assess random effect model fit (Burnham and Anderson, 2004). These models were: (1) a random effects structure that only allowed the intercepts to vary by participant; (2) a random effects structure that allowed both the intercepts and slopes (i.e., main effects) to vary by participant and; 3) a random effects structure that allowed the intercepts, slopes, and interactions between slopes to vary by participant. The AIC with the lowest value indicates the best-fitting model (Burnham and Anderson, 2004), with a difference of 10+ units demonstrating considerable evidence for a superior fit. The three random effect structures had AICs of 8224.31, 7566.23, and 7629.04, respectively, providing strong evidence that the random effects structure allowing both the slope and intercepts to vary by participant fits best.

Subsequently, the fixed effects were added to the random effect structure to examine any effects of the predictor variables (i.e., graph type and relationship type) on the outcome variable (participant performance). This was done by comparing the fit of two models, again using the AIC. The first model contained the main effects of both graph type and relationship type only. (Both predictors were included simultaneously based on two considerations: (a) both are experimentally manipulated variables with associated hypotheses, and (b) atheoretically testing all possible models would needlessly increase the likelihood of spurious results.) The second model tested was identical to the first with the addition of an interaction coefficient between graph type and relationship type.

**FIGURE 3:**
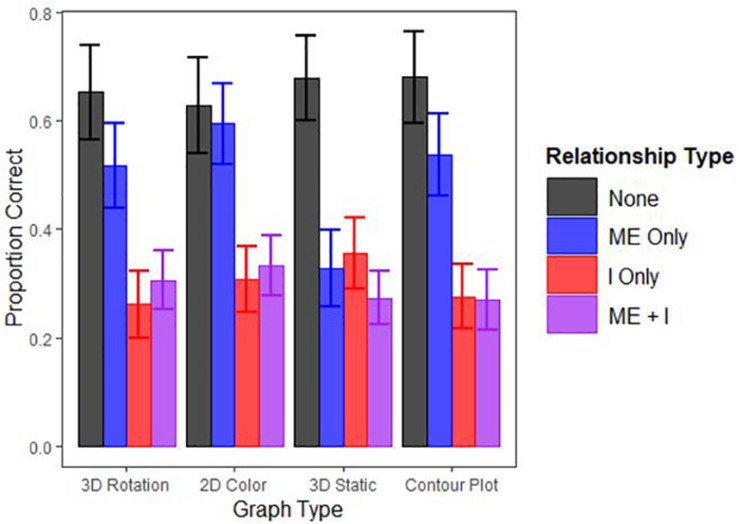
Proportion of questions answered correctly by Graph and Relationship Type. “ME” indicates a main effect and “I” indicates an interaction effect (i.e., “ME”+“I” is a main effect and an interaction. Note error bars represent 95% confidence intervals.

When the AICs of these models were compared, the second model containing both main effects and the interaction had a considerably lower AIC (AIC = 7456.42) than did the simpler model lacking the interaction term (AIC = 7494.61) suggesting the more complex model fits the data better. Figure 3 displays participants’ overall performance across the different graph types by relationship type and Table 1 displays the model coefficients. Performance decreased as a function of relationship complexity, supporting Hypothesis 2. Performance generally did not vary across graph type, however, with the exception that the interpretation of main effects was significantly worse on the static 3D scatterplots and this negative effect was mitigated when participants could rotate the 3D scatterplot. Thus, graph type did not affect performance in most cases, contrary to Hypothesis 1. The results suggest that the efficacy of a particular graph format may, in some situations, depend on the type of relationship it is intended to communicate, and specifics of this conjecture should be pursued in further research.

**TABLE 1:** The effects of graph type, relationship type, and their interaction on participant performance.

**Predictors**	**Fixed effects**	**Estimate**	**Std. Error**	***p***
–	Intercept	0.37	0.16	0.017
Graph types	3D Static	–1.1	0.17	< 0.001^∗^
	3D Rotatable	–0.31	0.17	0.075
	Contour plot	–0.22	0.17	0.19
Relationship	No effect	0.15	0.23	0.509
types	Interaction only	–1.19	0.21	< 0.001^∗^
	Main effect and interaction	–1.07	0.2	< 0.001^∗^
Interactions	3D Static * No relationship	1.32	0.24	< 0.001^∗^
	3D Static * Main effect and	0.81	0.24	< 0.001^∗^
	interaction			
	3D Static * Interaction only	1.31	0.24	< 0.001^∗^
	3D Rotatable * No relationship	0.42	0.24	0.083
	3D Rotatable * Main effect and	0.19	0.23	0.419
	interaction			
	3D Rotatable * Interaction only	0.08	0.24	0.728
	Contour Plot * No relationship	0.45	0.24	0.059
	Contour Plot * Main effect and	–0.08	0.23	0.73
	interaction			
	Contour Plot * Interaction only	0.07	0.24	0.773

*^∗^indicates a statistically significant effect.*

## Discussion

Interpreting interactions in data is difficult, as many research methods and statistics instructors know well, yet there is scant work directly addressing how to improve this issue. Conventional wisdom is that graphical representations help in recognizing interaction effects. Kosslyn’s (2006) eight principles can serve as a useful foundation for basic graph construction, but these principles are insufficient when building a graph to portray higher dimensions. There are unresolved disagreements at this level about fundamental properties of visual displays and the efficacy with which different graphs convey the requisite information. It is also imperative to remember that the most effective methods of communicating complex results may be emerging methods that leverage modern technologies and graphing methods. An empirical and experimental approach to graph optimization can help identify better methods and thereby help to make those methods a part of standard research practices.

The present study found that the lack of any effect is decently perceivable by people, and interactions (either alone or with a main effect) remain difficult to perceive across all graph formats used in the present study (Table 1). Main effects are relatively difficult to perceive in static 3D graphs, relative to other graph formats (see Figure 3). Unfortunately, static 3D graphs seem to also be a currently popular presentation option because it does not require dynamic- or color-enabled media. An implication of the present study, however, is that 3D static graphs should be discarded in favor of either their 2D counterparts or, format permitting, their 3D rotatable variants. Further study is warranted for graphs of more complex relationships, for which the results were inconclusive.

The present study points toward a few future research directions. Overall performance, though above chance, was poor. Introductory psychology undergraduates (and perhaps people in general) are ill-equipped to identify complex continuous relationships in graphs. Graphical literacy should be a concern for educators and employers across all fields that work with data. The poor overall performance in the current study also limits the extent to which broader effects of graph and relationship type on statistical interpretation can be measured. Assessing a more graph-savvy population (e.g., researchers in a quantitative field) could reveal clearer effects with greater variability in performance ability. Additionally, there is clear potential for creating and implementing novel graph types that are geared to facilitate the interpretation of interactions (e.g., a profiler plot).

## Data Availability Statement

The datasets generated for this study are available on request to the corresponding author.

## Ethics Statement

The studies involving human participants were reviewed and approved by the Kansas State University Institutional Review Board (IRB). The patients/participants provided their written informed consent to participate in this study.

## Author Contributions

JH contributed to the conception and design of the work, data collection, data analysis and interpretation, and drafting of the manuscript. CG contributed to the conception and design of the work, and collection of the data. GB contributed to the design, interpretation of the data, and critical revision of the manuscript.

## Conflict of Interest

The authors declare that the research was conducted in the absence of any commercial or financial relationships that could be construed as a potential conflict of interest.

## Footnotes

1 https://plot.ly/
